# Diabetes in Iran: Prospective Analysis from First Nationwide Diabetes Report of National Program for Prevention and Control of Diabetes (NPPCD-2016)

**DOI:** 10.1038/s41598-017-13379-z

**Published:** 2017-10-18

**Authors:** Alireza Esteghamati, Bagher Larijani, Mohammad Haji Aghajani, Fatemeh Ghaemi, Jamshid Kermanchi, Ali Shahrami, Mohammad Saadat, Ensieh Nasli Esfahani, Morsaleh Ganji, Sina Noshad, Elias Khajeh, Alireza Ghajar, Behnam Heidari, Mohsen Afarideh, Jeffrey I. Mechanick, Faramarz Ismail-Beigi

**Affiliations:** 1 Endocrinology and Metabolism Research Center (EMRC), Vali-Asr Hospital, School of Medicine, Tehran University of Medical Sciences, Tehran, Iran; 2 Diabetes Researcher Center, Endocrinology and Metabolism Research Institute, Tehran University of Medical Sciences, Tehran, Iran; 3 Shahid Beheshti University of Medical Sciences, Tehran, Iran; 4 Diabetes Program, Deputy of Health, Ministry of Health and Medical Education (MOHME), Tehran, Iran; 5 Deputy of Curative Affairs, Ministry of Health and Medical Education (MOHME), Tehran, Iran; 6 Division of Endocrinology, Diabetes, and Bone Disease, Icahn School of Medicine at Mount Sinai, New York, New York United States; 7 Department of Medicine, Biochemistry, Physiology and Biophysics, Division of Clinical and Molecular Endocrinology, Case Western Reserve University, Cleveland, Ohio United States

## Abstract

We estimated proportions of different types of diabetes, comorbidities, treatment (the use of oral glucose-lowering agents and insulin), control (hyperglycemia, dyslipidemia and hypertension) and chronic microvascular and macrovascular complications among people with diabetes presenting to the tertiary-care academic diabetes outpatient clinics in Iran. This study is the prospective analysis of data (*n* = 30,202) from the registry of university-affiliated adult outpatient diabetes clinics in the country during 2015–2016. The proportions of type 1 diabetes, types 2 diabetes, and other types of diabetes were 11.4%, 85.5%, and 1.3%, respectively. The frequencies of drug-naivety, use of oral agents, insulin monotherapy and insulin combination therapy were 2.9%, 60.5%, 11.5%, and 25.1%, respectively. Around 13.2%, 11.9% and 43.3% of patients with diabetes had controlled hyperglycemia, hyperlipidemia and hypertension, respectively. The proportions of retinopathy, nephropathy, peripheral neuropathy, diabetic foot, and ischemic heart disease were 21.9%, 17.6%, 28.0%, 6.2%, and 23.9%, respectively. Despite the wide availability of medications and insulin coverage in Iran, the estimated national control of hyperglycemia, hyperlipidemia and hypertension (especially for young men and old women) remains subpar. The present study further suggests that the frequencies of chronic vascular complications among patients with diabetes are relatively high in Iran.

## Introduction

In 2013, at least 382 million adults had diabetes (all types) worldwide^1^, and this number rose to 422 million by 2014^2^. The United States (US) witnessed a 4.5% annual growth rate of patients with diabetes during 1998–2008, before the prevalence of diabetes leveled off with no significant change from 2008 to 2012^3^. The 2013 International Federation of Diabetes (IDF) Atlas for Diabetes ranked the Middle East and North Africa (MENA) with the highest worldwide prevalence of diabetes at 10.9%^1^. The MENA region is projected to have the second highest global growth rate in terms of the number of affected individuals with diabetes, with 96.2% by 2035^1^. In Iran, the fourth round of the periodic National Survey of Risk Factors for Non-communicable Diseases project in 2011 (SuRFNCD-2011) estimated the national prevalence of diabetes at 11.4% of the adult population in Iran, showing a 35% increase from the inaugural report in 2005^4^. This followed a doubling in the rate of diabetes between 1999 and 2007 in the country^5^. While approximately 4.5 million adult people were living with diabetes in Iran in 2011, over a quarter of this population were not previously diagnosed^4^; and it is estimated that by the year 2030, 9.2 million Iranian individuals will have diabetes^6^. This continuous and significant increase in the prevalence of diabetes reflects the high burden of disease in Iran, especially when considering the impact of diabetes-related complications^7–11^.

In line with the earnest global initiatives to reduce burden of diabetes, the National Program for Prevention and Control of Diabetes (NPPCD) of Iran has made great strides towards providing effective prevention of general population against diabetes and sustained care for patients diagnosed with diabetes from 2004 onwards^12,13^. Despite the continuous efforts of the NPPCD to provide local, regional, and subnational estimates of diabetes^13^, the current status of type-specific status, natural history, comorbidities, quality and accessibility of care, indicators of control, diabetes-related complications, and burden of diabetes remain poorly characterized among patients with diabetes in Iran. By comparison, the 2009 Catalyst to Better Diabetes Care Act mandated the Center for Disease Control and Prevention (CDC) to publish biannual “Diabetes Report Cards” for patients with diabetes in the US^14^.

The purpose of the present study was to provide, for the first time, comprehensive information on various aspects of diabetes care in Iran, including the proportions of different types of diabetes (type 1 diabetes [T1D], type 2 diabetes [T2D], and other types of diabetes), associated comorbidities/cardiometabolic risk factors, indicators of access to care, control of disease, and chronic vascular diabetes-related complications among the population of adult people with diabetes referred to tertiary-care academic diabetes outpatient clinics across the country. To achieve this, comprehensive data from the national database of NPPCD were accessed, aggregated, and pooled for the analysis.

## Research Design and Methods

### Data source and registry

The data source for the current study included the NPPCD database of adult people with diabetes who presented to academic tertiary-care diabetes outpatient clinics across the country between December 1, 2015 and June 31, 2016 (NPPCD-2016). A detailed description of the NPPCD, its characteristics, goals, and how it operates to reduce the burden of diabetes in Iran has been recently published^13^. The inclusion criteria included physician-adjudicated diagnosis for different types of diabetes, made during the period of survey (i.e., December 1, 2015–June 31, 2016) on consecutive patients who attended 71 diabetes outpatient clinics from 55 medical universities located across 31 provinces of country. The host cities for the implementation of the NPPCD-2016 included the capital cities of included provinces. During the initial registry enrollment period, 31,096 people with different types of diabetes were enrolled. The types of diabetes were clinically determined by the physicians at each clinic and categorized as T1D, T2D, and other types of diabetes (including other, hybrid, monogenic diabetes, secondary diabetes, latent autoimmune diabetes of adults [LADA], atypical, unknown, and missing type). Prior to the analysis, patients aged <18 years old (*n* = 352, around 1% of total population) and those with missing data on the demographic, clincial, and biochemical characteristics (*n* = 542, 1.7% of total population) were excluded from the database. Consequently, data from 30,202 people with different types of diabetes were included for analysis. Herein, after excluding cases with incomplete data (*n* = 542, 1.7%) for at least one of the study outcomes (except for serum lipid measures, for which random subsamples of ~30% of the target population referring to each of the university-affiliated diabetes outpatient clinics were prespecified [i.e., priori designed] to be obtained in the study protocol), we conducted the complete case analysis on data from the rest of patients. These included individuals in the complete case analysis represented individuals with fully recorded data for all of the non-lipid study variables and outcomes. All reported methods were carried out in accordance with the latest revision of the Declaration of Helsinki 1964. The ethics committees of the MOHME and local universities approved of the study protocol. Prior to enrollment, each of the included patients provided written informed consents to participate in the study.

### Physical examinations

Physical examinations were performed by trained interviewers and included measurement of weight, height, waist circumference, and blood pressure. Weight was measured on a portable digital scale with patients wearing light clothing and was recorded to the nearest 0.1 kg. An inflexible measurement tape was used to measure height while the patient standing still, with no shoes or socks on, and was recorded with a precision of 0.1 cm. Body mass index (BMI) was calculated as weight (kg) divided by height (m^2^). After resting for at least 10 min, three blood pressure measurements, 5 min apart, were taken by trained health staff using the calibrated Omron M7 digital sphygmomanometers (Hoofddorp, The Netherlands) with the appropriate sized cuff covering at least 80% of the right arm. The first reading was discarded and mean values for systolic and diastolic blood pressures (SBP and DBP, respectively) were determined by averaging the second and third readings.

### Laboratory evaluations

Patients were instructed to fast overnight for 12–14 h prior to blood sampling the following morning. At each center, 10 mL of venous blood was drawn and collected from each patient, sampled in cold biochemistry tubes (4–8 °C), and sent within 4 h to representative collaborating laboratories. Samples were then immediately centrifuged (1500 Rmp for 10 min at standard room temperature: 21 °C) and the extracted serum was used for laboratory evaluations. Fasting plasma glucose (FPG) and 2-h postprandial glucose (2hPPG) were measured with enzymatic calorimetric methods using the glucose oxidase test. High-performance liquid chromatography was used to measure glycated hemoglobin (A1C, DS5 Pink kit; Drew, Marseille, France). Serum concentrations of triglycerides, total cholesterols, low-density lipoprotein cholesterol (LDL-C), and high-density lipoprotein cholesterol (HDL-C) were determined by enzymatic methods. All measurements were performed using quality-controlled commercially available kits (Pars Azmun, Karaj, Iran) provided and distributed by the central reference laboratory (Tehran, Iran). Random samples were also sent to the central laboratory to check for the accuracy of measurements and, if significant deviations were flagged, the results were discarded.

### Definitions of comorbidities and treatment targets

The status of demographic risk factors (i.e., age and duration of diabetes), indictors of glycemic, lipid and blood pressure control, and common comorbidities (self-identified history of smoking, obesity [defined for Iranians as BMI ≥25 kg/m^2^] and hypertension [defined as SBP ≥140 mm Hg or DBP ≥90 mm Hg, previous history of hypertension, use of prescribed antihypertensive medications or a “yes” answer to the following question: “*have you ever been told by a physician that you have hypertension?*”), along with use of oral glucose-lowering agents and/or insulin, as well as chronic diabetes-related vascular complications were assessed. ABC (A1C, blood pressure, cholesterol [LDL-C]) control target thresholds were preset according to the American Diabetes Association recommended cutoffs on glycemic, lipid, and blood pressure indices^15,16^: The desirable ranges for preprandial capillary plasma glucose (i.e., FPG) and peak postprandial plasma glucose (i.e., 2hPPG) were set as 80 mg/dL (4.4 mmol/L) <FPG <130 mg/dL (7.2 mmol/L) and 2hPPG <180 mg/dL (10.0 mmol/L). Uncontrolled glycemia was defined as the presence of A1C levels >7.0% (53 mmol/mol). The preset targets for indices of lipid control were triglyceride levels <150 mg/dL (1.7 mmol/L), total cholesterol levels <200 mg/dL (5.2 mmol/L), LDL-C level <100 mg/dL (2.6 mmol/L); and HDL-C levels >50 mg/dL (1.3 mmol/L) and 40 mg/dL (1.0 mmol/L) in women and men, respectively. In patients with different types of diabetes, the preset targets for the control of blood pressure were determined at SBP ≤140 mm Hg and DBP ≤90 mm Hg.

To identify diabetes-related chronic microvascular complications, *the International Classification of Diseases*, *Tenth Revision* (*ICD-10*) was used. The specific codes used were: E10.2, E11.2, E12.2, E13.2 and E14.2 for diabetic nephropathy; E10.3, E11.3, E12.3, E13.3 and E14.3 for diabetic retinopathy; E10.4, E11.4, E12.4, E13.4 and E14.4 for diabetic neuropathy; and E10.5, E11.5, E12.5, E13.5 and E14.5 for diabetic foot. Ischemic heart disease (IHD) was defined on the grounds of any of the following: the physician-adjudicated nonfatal coronary artery disease (inclusive of angina pectoris, coronary insufficiency, acute coronary syndrome, myocardial infarction, coronary artery bypass graft surgery, coronary angioplasty, percutaneous coronary intervention, or other revascularization procedures); positive findings in noninvasive tests including the myocardial perfusion scan, exercise tolerance test, transthoracic echocardiography, or multidetector computed tomography coronary angiography that prompted physicians to start anti-ischemic treatment; or a “yes” answer to any of the following questions: “*have you ever been told by a cardiologist that you have a coronary artery disease/disease of heart vessels”?* or “*have you ever been told by a cardiologist that you should take medication for your heart vessels”*?

### Statistical methods

Statistical analysis was performed using the STATA software version 12 for Windows (Stata Corp., College Station, TX, US). Based on the results of normality tests (i.e., Shapiro-Wilks test), continuous variables (i.e., age, duration of disease, SBP DBP, glycemic indices, and lipid indices) are presented either as mean ± standard deviation (SD)/standard error of the mean (S.E.M) or median (interquartile range [IQR]). Categorical outcomes (i.e., different types of diabetes, comorbidities, medication use, meeting the preset glycemic, blood pressure and lipid control targets, and chronic diabetes-related microvascular and macrovascular complications) are demonstrated as proportions (95% confidence interval [95% CI]) or mean ± S.E.M. The proportions of these categorical variables were classified according to age and duration of disease. Trends of proportions over the classes of age and duration of diabetes were tested for statistical significance using the non-parametric Cochran-Armitage test. Differences in proportions across the binary characteristics (i.e., gender) were assessed using a design-based Chi-square test. To compare continuous data between the binary variables (i.e., gender), design-based parametric independent *t*-test or non-parametric Mann-Whitney *U* Test were used, as appropriate. A two-sided *p* value < 0.05 was considered necessary to reject the null hypothesis.

## Results

### Baseline characteristics

A total of 30,202 people with diabetes who fulfilled the eligibility criteria were included. The median (IQR) age of the study population was calculated at 59.0 (52.0 to 66.0) years old for men and 57.0 (50.0 to 64.0) years old for women (*p* value < 0.001). The median diagnostic age at the time of diabetes onset was 50.0 (IQR: 40.0 to 58.0) years old for men and 47.0 (IQR: 39.0 to 55.0) years old for women (*p* value < 0.001), corresponding to overall median (IQR) duration of diabetes of 8.0 (4.0 to 14.0) years (median [IQR]: 7.0 [3.0 to 14.0] years in men vs. 8.0 [4.0 to 14.0] years in women, *p* value < 0.001).

### Proportions of diabetes types and comorbid conditions

In the entire population cohort, the proportions (95% CIs) of T1D, T2D, and other types of diabetes were 11.4% (11.0% to 11.8%), 85.5% (85.1% to 85.9%), and 1.3% (1.2% to 1.4%), respectively (Table 1). The proportion (95% CI) of patients with gestational diabetes mellitus was 1.8% (1.7% to 1.9). However, data of patients with gestational diabetes mellitus were excluded from the pooled analysis. Unlike all other forms of diabetes, gestational diabetes mellitus is a transient disease and the spectrum of treatments, and co-morbidities and is very different. Thus, focusing on permanent diabetes in this publication provided a clearer picture of diabetes in Iran. There was a significant difference in gender-specific proportions of diabetes types, particularly for T1D, namely 10.6% (95% CI: 10.2% to 11.0%) for women vs. 13.0% (95% CI: 12.3% to 13.7%) for men (*p* value < 0.001, Table 1

**Table 1:** Comorbidities, types of diabetes and treatments among adult clinically-registered people with diabetes in Iran.

Gender	Age (years)	Frequency	Smoking*^ɸǂ^	Obesity*^ɸǂ^	Hypertension*^ɸǂ^	Type of Diabetes*^ɸǂ^			Treatment*^ɸǂ^			
						Type 1	Type 2	Other	Drug-Naïve	Oral Agent	Insulin	Combination
		Number (%)	Proportion	Proportion	Proportion	Proportion	Proportion	Proportion	Proportion	Proportion	Proportion	Proportion
			95% CI	95% CI	95% CI	95% CI	95% CI	95% CI	95% CI	95% CI	95% CI	95% CI
Women	≤44	2,729 (9.0%)	1.40%	43.30%	15.40%	18.40%	60.20%	1.90%	9.20%	45.70%	24.90%	20.20%
			1–1.8	41.4–45.2	14–16.8	16.9–19.9	58.4–62	1.4–2.4	8.1–10.3	43.8–47.6	23.3–26.5	18.7–21.7
	45 to 64	12,952 (42.9%)	2.20%	54.20%	38.30%	8.80%	90.00%	1.10%	1.60%	63.60%	8.30%	26.50%
			1.9–2.5	53.3–55.1	37.5–39.1	8.3–9.3	89.5–90	0.9–1.3	1.4–1.8	62.8–64.4	7.8–8.8	25.7–27.3
	≥65	4,417 (14.6%)	2.60%	50.80%	52.10%	10.80%	88.30%	0.80%	1.10%	61.70%	11.30%	25.90%
			2.1–3.1	49.3–52.3	50.6–53.6	9.9–11.7	87.4–89.2	0.5–1.1	0.8–1.4	60.3–63.1	10.4–12.2	24.6–27.2
	Total	20,098 (66.5%)	2.20%	52.00%	38.20%	10.60%	85.60%	1.10%	2.50%	60.70%	11.20%	25.50%
			2–2.4	51.3–52.7	37.5–38.9	10.2–11	85.1–86.1	1–1.2	2.3–2.7	60–61.4	10.8–11.6	24.9–26.1
Men	≤44	1,248 (4.1%)	12.30%	36.60%	18.50%	25.50%	71.40%	3.00%	8.00%	50.50%	18.30%	23.20%
			10.5–14.1	33.9–39.3	16.3–20.7	23.1–27.9	68.9–73.9	2.1–3.9	6.5–9.5	47.7–53.3	16.2–20.4	20.9–25.5
	45 to 64	5,740 (19.0%)	14.90%	44.30%	39.30%	10.60%	87.60%	1.70%	3.40%	61.30%	10.80%	24.50%
			14–15.8	43–45.6	38–40.6	9.8–11.4	86.7–88.5	1.4–2	2.9–3.9	60–62.6	10–11.6	23.4–25.6
	≥65	3,116 (10.3%)	11.90%	41.10%	50.90%	12.30%	86.60%	1.20%	2.30%	61.60%	12.10%	24.00%
			10.8–13	39.4–42.8	49.1–52.7	11.1–13.5	85.4–87.8	0.8–1.6	1.8–2	59.9–63.3	11–13.2	22.5–25.5
	Total	10,104 (33.5%)	13.60%	42.40%	40.30%	13.00%	85.30%	1.70%	3.60%	60.00%	12.20%	24.20%
Total		30,202 (100%)	12.9–14.3	41.4–43.4	39.3–41.3	12.3–13.7	84.6–86	1.4–2	3.2–4	59–61	11.6–12.8	23.4–25
			6.00%	48.80%	38.90%	11.40%	85.50%	1.30%	2.90%	60.50%	11.50%	25.10%
			5.7–6.3	48.2–49.4	38.4–39.4	11–11.8	85.1–85.9	1.2–1.4	2.7–3.1	59.9–61.1	11.1–11.9	24.6–25.6

95% CI: 95% confidence interval. *Denotes statistically significant difference between men and women (*p* value < 0.05). ^ɸ^Denotes statistically significant difference across the age groups of women (*p* value < 0.05). ^Ŧ^Denotes statistically significant difference across the age groups of men (*p* value < 0.05).

). When considering all types of diabetes, women comprised 65% of the population under study.

The frequencies of cardiometabolic risk factors were significantly different for men and women with diabetes, with obesity being a more predominant trait among women (52.0% [95% CI: 51.3% to 52.7%] vs. 42.4% [95% CI: 41.4% to 43.4%], *p* value < 0.001) and hypertension a more frequent comorbid condition in men (40.3% [95% CI: 39.3% to 41.3%] vs 38.2 [95% CI: 37.5% to 38.9%], *p* value < 0.001, Table 1). The overall frequencies of patients with diabetes, with or without hypertension, were calculated at 38.9 (95% CI: 38.4% to 39.4%) and 61.1% (95% CI: 59.5% to 60.7%), respectively. In total, 77.9% (95% CI: 77.5% to 78.4%) of the entire population cohort achieved preset targets for the control of blood pressure. Among patients with hypertension, the proportions of controlled and uncontrolled hypertension based on the preset blood pressure targets were 43.3% (95% CI: 42.5% to 44.3%) and 56.7% (95% CI: 55.8% to 57.6%), respectively. The average SBP/DBP values were 120.7 ± 18.1/74.2 ± 9.2 mm Hg in normotensive patients and 142 ± 28.1/87.5 ± 15.3 mm Hg in patients with hypertension (*p* values of the difference for both SBP and DBP <0.001). Smoking was also more prevalent among men with diabetes (13.6% [12.9% to 14.3%] for men compared to 2.2% [95% CI: 2.0% to 2.4%] for women, *p* value < 0.001, Table 1).

All patients with hypertension or obesity (gender-specific *p* for trend values for both risk factors <0.001), and women who smoke tobacco (*p* for trend value <0.001) were more likely to belong to the older age groups; whereas older men smoked with less frequency than their younger counterparts (*p* for trend value <0.001). The index-specific status of comorbid risk factors in patients with T2D is presented in the Supplementary Material.

### National access to the diabetes care

The overall proportions (95% CIs) of drug-naivety (i.e. inclusive of nontreatment, diet control, or lifestyle modification), oral glucose-lowering medication use, insulin monotherapy and combination therapy by insulin plus oral glucose-lowering drugs were 2.9% (2.7% to 3.1%), 60.5% (59.9% to 61.1%), 11.5% (11.1% to 11.9%) and 25.1% (24.6% to 25.6%), respectively (Table 1).

**Figure 1:**
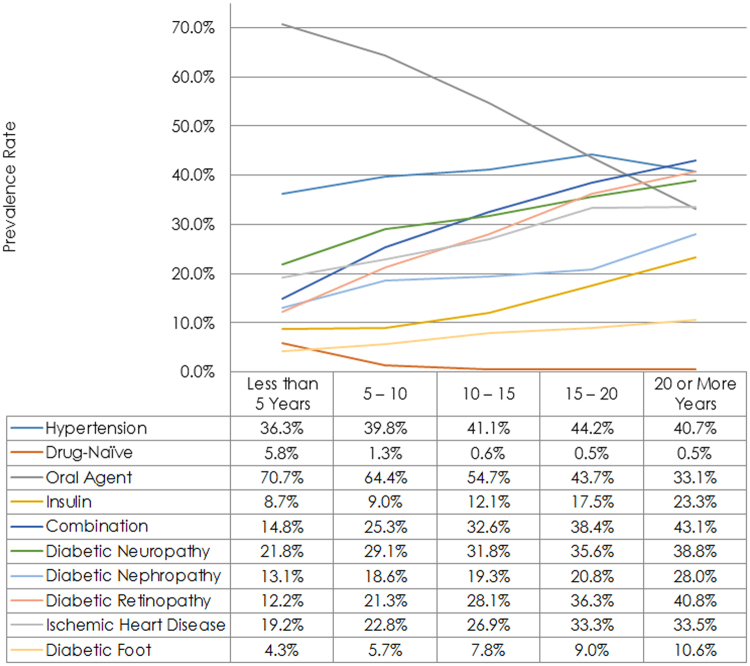
Hypertension, treatments and diabetes-related microvascular complications by duration of disease among the clinically-registered adult patients with diabetes in Iran.

In total, smaller proportions of drug-naive patients and patients receiving insulin monotherapy were observed in older age classes (gender-specific *p* for trend values <0.001). In contrast, older patients were more frequently managed by oral glucose-lowering medications outside or inside the combination therapy with insulin (*p* for trend values <0.001) (Table 1). Men with an earlier diagnosis of diabetes had significantly lower frequencies of drug-naivety and use of oral hyperglycemic agents; and higher frequency of insulin monotherapy and combination therapy by insulin plus oral agents (*p* for trend values <0.001, Fig. 1). Breakdown of patients with T2D according to the status of treatment is demonstrated in the Supplementary Material.

Analysis of treatment targets for specific and overall control of glycemic and lipid indices is demonstrated in Table 2. Mean ± standard error of the mean (S.E.M) values of FPG, 2hPPG, A1C, triglycerides, total cholesterol, LDL-C and HDL-C were 171.8 ± 0.4 mg/dL, 239.8 ± 0.5 mg/dL, 8.00 ± 0.01%, 160.9 ± 1.0 mg/dL, 175.2 ± 0.4 mg/dL, 98.1 ± 0.3 mg/dL, and 43.3 ± 0.1 mg/dL, respectively, for the entire study population. A superior global glycemic control (i.e., of all glycemic indices) was present among women (mean ± S.E.M: 14.0 ± 0.5% vs. 11.6 ± 0.6%, *p* value < 0.001), while hyperlipidemia was better controlled among men (mean ± S.E.M: 16.9 ± 1.1% vs. 9.5 ± 0.7%, *p* value < 0.001, Table 2

**Table 2:** Proportions of meeting the preset glycemic and lipid targets based on standard biochemical measures among adult clinically-registered people with diabetes in Iran.

Gender	Age (years)	Frequency	FPG (mg/dL)*^ǂ^		2hPPG (mg/dL)*^ɸǂ^		A1C (%)*^ɸǂ^		Triglyceride (mg/dL)*^ǂ^		Total Cholesterol (mg/dL)*^ǂ^		LDL-C (mg/dL)*^ǂ^		HDL-C (mg/dL)*		All Glycemic Indices Control*^ɸ^		All Lipid Indices Control*	
			% of Control	SEM	% of Control	SEM	% of Control	SEM	% of Control	SEM	% of Control	SEM	% of Control	SEM	% of Control	SEM	% of Control	SEM	% of Control	SEM
		Number (%)	Mean	SEM	Mean	SEM	Mean	SEM	Mean	SEM	Mean	SEM	Mean	SEM	Mean	SEM				
Women	≤44	2,729 (9.0%)	37.30%	1.80%	42.90%	2.10%	52.60%	1.90%	58.70%	3.20%	72.20%	2.90%	54.50%	3.20%	28.30%	2.90%	23.70%	1.60%	10.50%	2.00%
			162.7	1.4	214.7	1.8	7.60%	0.01%	163.8	3.8	179	1.4	102.4	1.1	44.8	0.4				
	45 to 64	12,952 (42.9%)	27.20%	0.80%	26.30%	0.80%	41.40%	0.80%	55.20%	1.30%	73.00%	1.10%	57.70%	1.30%	27.40%	1.10%	12.40%	0.60%	9.00%	0.70%
			176.5	0.6	242	1.5	8.10%	0.01%	164.2	1.4	179.2	0.6	99.4	0.4	44.8	0.2				
	≥65	4,417 (14.6%)	31.30%	1.40%	26.90%	1.20%	45.90%	1.50%	57.30%	2.20%	74.30%	2.00%	57.60%	2.20%	27.70%	2.00%	12.80%	1.00%	10.30%	1.40%
			167.2	1	238.3	1.3	7.90%	0.02%	160.3	2.2	178.4	1	100.1	0.8	44.6	0.3				
	Total	20,098 (66.5%)	29.50%	0.60%	28.70%	0.30%	43.90%	0.70%	56.10%	1.10%	73.20%	1.00%	57.30%	1.10%	27.50%	1.00%	14.00%	0.50%	9.50%	0.70%
			172.6	0.5	237.5	0.6	8.00%	0.01%	163.3	1.2	179	0.5	99.8	0.4	44.8	0.1				
Men	≤44	1,248 (4.1%)	26.30%	2.40%	24.70%	2.40%	39.70%	2.70%	55.80%	4.30%	76.30%	3.70%	59.60%	4.20%	54.00%	4.30%	10.30%	1.70%	17.50%	3.30%
			188.2	2.4	258.8	3	8.40%	0.01%	170.8	5.5	174.7	1.9	99.2	1.6	42.3	0.6				
	45 to 64	5,740 (19.0%)	30.10%	1.20%	25.10%	1.10%	44.10%	1.30%	58.40%	2.00%	79.40%	1.60%	62.30%	1.90%	47.10%	2.00%	11.70%	0.80%	16.10%	1.50%
			171	0.9	243.8	1.2	8.00%	0.01%	161.2	2.2	168.7	0.9	95.3	0.7	40.2	0.2				
	≥65	3,116 (10.3%)	35.70%	1.70%	25.80%	1.20%	46.70%	1.80%	69.60%	2.50%	84.90%	2.00%	66.20%	2.60%	45.80%	2.70%	11.90%	1.10%	18.10%	2.10%
			161.7	1.2	240.3	1.5	7.90%	0.01%	139.6	2.6	161.5	1.2	91.7	0.9	40.3	0.3				
	Total	10,104 (33.5%)	31.40%	0.90%	25.30%	0.80%	44.40%	1.00%	61.50%	1.50%	80.70%	1.20%	63.20%	1.50%	47.60%	1.50%	11.60%	0.60%	16.90%	1.10%
			170.3	0.7	244.6	0.9	8.00%	0.01%	155.8	1.6	167.3	0.7	94.6	0.5	40.5	0.2				
Total		30,202 (100%)	30.10%	0.50%	27.60%	0.50%	44.10%	0.60%	57.80%	0.90%	75.60%	0.80%	59.20%	0.90%	34.10%	0.90%	13.20%	0.40%	11.90%	0.60%
			171.8	0.4	239.8	0.5	8.00%	0.01%	160.9	1	175.2	0.4	98.1	0.3	43.3	0.1				

SEM: standard error of the mean. *Denotes statistically significant difference between men and women (*p* value < 0.05). ^ɸ^Denotes statistically significant difference across the age groups of women (*p* value < 0.05). ^Ŧ^Denotes statistically significant difference across the age groups of men (*p* value < 0.05).

).

Women in higher age classes showed a significant decline in 2hPPG (*p* < 0.001) and A1C (*p* < 0.001), compared to younger women. By contrast, women had comparable control of hyperlipidemia across different age classes (*p* for trend value for triglycerides, total cholesterol, LDL-C and HDL-C = 0.0388, 0.689, 0.084 and 0.583, respectively). Furthermore, younger men had compromised control of all glucose and lipid indices, except HDL-C (better control for younger men, *p* for trend = 0.002), namely FPG (*p* for trend <0.001), 2hPPG (*p* for trend <0.001), A1C (*p* for trend <0.001), triglycerides (*p* for trend <0.001), total cholesterol (*p* for trend <0.001), and LDL-C (*p* for trend <0.001), compared to their older counterparts (Table 2). Global control of glycemic treatment targets in women with different types of diabetes was better achieved in the subset of patients with a lower duration of disease (<10 years) compared to patients with longer course of diabetes (>20 years, *p* for trend value for global glycemic control <0.001). Men with fewer years since their diagnosis of diabetes also achieved better control of FPG (*p* for trend value <0.001), but not of 2hPPG (*p* for trend value = 0.175) and A1C (*p* for trend value = 0.097). Men with longer duration of diabetes had significantly better overall control of lipid indices compared to patients with a more recent diagnosis (*p* for trend values for, triglycerides, total cholesterol, LDL-C and HDL-C <0.001, <0.001, = 0.001 and = 0.001, respectively). However, women with different types of diabetes had comparable control of hyperlipidemia across the higher and lower classes of diabetes duration (*p* for trend values for triglycerides, total cholesterol, LDL-C and HDL-C = 0.542, 0.276, 0.102 and 0.200, respectively). Supplementary Material displays the index-specific status of achieving the preset glycemic and lipid treatment targets among patients with T2D.

### Current Status of Chronic Vascular Complications

Total percentages (95% CI) of chronic vascular complications were 21.9% (21.4% to 22.4%), 17.6% (17.2% to 18.0%), 28.0% (27.5% to 28.5%), 6.2% (5.9% to 6.5%), and 23.9% (23.4% to 24.4%) for diabetic retinopathy, diabetic nephropathy, diabetic peripheral neuropathy, diabetic foot ulcers, and ischemic heart disease due to diabetes, respectively (Table 3). A significant gender preference was observed for diabetic nephropathy (16.70% [95% CI: 16.18% to 17.22%] for women vs. 19.40% [95% CI: 18.63% to 20.17%] for men, *p* value < 0.001), diabetic peripheral neuropathy (29.20% [95% CI: 28.57% to 29.83%] for women vs. 25.70% [95% CI: 24.85% to 26.55%] for men, *p* value < 0.001), diabetic foot (5.20% [95% CI: 4.89% to 5.51%] for women vs. 8.30% [95% CI: 7.76% to 8.84%] for men, *p* value < 0.001) and ischemic heart disease (23.20% [95% CI: 22.62% to 23.7%] for women vs. 25.10% [95% CI: 24.25% to 25.95%] for men, *p* value < 0.001). However, the fraction of patients with diabetic retinopathy was comparable between women and men (21.80% [95% CI: 21.23% to 22.37%] and 21.90% [95% CI: 21.09% to 22.71%], respectively; *p* value = 0.471, Table 3

**Table 3:** Prevlance of chronic vascular complications among adult clinically-registered people with diabetes in Iran.

Gender	Age (years)	Frequency	Retinopathy^ɸǂ^	Nephropathy*^ɸǂ^	Neuropathy*^ɸ^ǂ	Ischemic Heart Disease*^ɸǂ^	Diabetic Foot*^ɸǂ^	Number of Microvascular Complications*^ɸǂ^			Any Complicationɸ^ǂ^
								1	2	3	
			Proportion	Proportion	Proportion	Proportion	Proportion	Proportion	Proportion	Proportion	Proportion
		Number (%)	95% CI	95% CI	95% CI	95% CI	95% CI	95% CI	95% CI	95% CI	95% CI
Women	≤44	2,729 (9.0%)	10.10%	11.00%	16.70%	8.20%	2.70%	18.70%	6.40%	2.10%	32.30%
			8.97–11.23	9.83–12.17	15.3–18.1	7.17–9.23	2.09–3.31	17.24–20.16	5.48–7.32	1.56–2.64	30.55–34.05
	45 to 64	12,952 (42.9%)	22.30%	16.50%	30.30%	22.60%	5.30%	30.60%	13.10%	4.10%	57.50%
			21.58–23.02	15.86–17.14	29.51–31.09	21.88–23.32	4.91–5.69	29.81–31.39	12.52–13.68	3.76–4.44	56.65–58.35
	≥65	4,417 (14.6%)	27.70%	20.50%	33.80%	34.40%	6.30%	33.30%	15.60%	5.80%	66.90%
			26.38–29.02	19.31–21.69	32.40–35.20	33.00–35.80	5.85–7.02	13.91–34.69	14.53–16.67	5.11–6.49	65.51–68.29
	Total	20,098 (66.5%)	21.80%	16.70%	29.20%	23.20%	5.20%	29.60%	12.70%	4.20%	56.20%
			21.23–22.37	16.18–17.22	28.57–29.83	22.62–23.78	4.89–5.51	28.97–30.23	12.24–13.16	3.92–4.48	55.51–56.89
Men	≤44	1,248 (4.1%)	10.80%	16.00%	17.20%	8.00%	5.40%	22.90%	7.00%	2.40%	38.00%
			9.08–12.52	13.97–18.03	15.11–19.29	6.49–9.51	4.15–6.65	20.57–25.23	5.58–8.42	1.55–3.25	35.31–40.69
	45 to 64	5,740 (19.0%)	22.30%	18.10%	25.20%	23.50%	8.40%	28.10%	12.40%	4.20%	55.80%
			21.22–23.38	17.10–19.10	24.08–26.32	22.40–24.60	7.68–9.12	26.94–29.26	11.55–13.25	3.68–4.72	54.52–57.08
	≥65	3,116 (10.3%)	25.60%	23.00%	29.90%	34.80%	9.30%	28.90%	15.70%	6.10%	65.60%
			24.07–27.13	21.52–24.48	28.29–31.51	33.13–36.47	8.28–10.32	27.31–30.49	14.42–16.98	5.26–6.94	63.69–67.27
	Total	10,104 (33.5%)	21.90%	19.40%	25.70%	25.10%	8.30%	27.70%	12.70%	4.60%	56.60%
			21.09–22.71	18.63–20.17	24.85–26.55	24.25–25.95	7.76–8.84	26.83–28.57	12.05–13.35	4.19–5.01	55,63–57.57
Total		30,202 (100%)	21.90%	17.60%	28.00%	23.90%	6.20%	29.00%	12.70%	4.30%	55.70%
			21.43–22.37	17.17–18.03	27.49–28.51	23.42–24.38	5.93–6.47	28.49–29.51	12.32–13.08	4.07–4.53	55.14–56.26

95% CI: 95% confidence interval. *Denotes statistically significant difference between men and women (*p* value < 0.05). ^ɸ^Denotes statistically significant difference across the age groups of women (*p* value < 0.05). ^Ŧ^Denotes statistically significant difference across the age groups of men (*p* value < 0.05).

).

Proportions of diabetic retinopathy, diabetic nephropathy, diabetic peripheral neuropathy, ischemic heart disease, and diabetic foot consistently increased in higher age groups of men and women (*p* for trend values for all complications in both men and women <0.001). The fraction of patients with one, two, or three chronic microvascular complications was similar among men and women. When microvascular and macrovascular complications of diabetes were considered together, 56.60% of men (95% CI: 55.63% to 57.57%) and 56.20% of women (95% CI: 55.51% to 56.89%) had at least one chronic vascular complication of diabetes (*p* value of the gender difference = 0.213, Table 3). Status of chronic microvascular and macrovascular complications in patients with T2D is described in the Supplementary Material.

## Discussion

The inaugural NPPCD national diabetes report (NPPCD-2016) addressed mounting questions concerning the present makeup of diabetes, including the proportions of different types of diabetes, associated cardiometabolic risk factors, control and access to diabetes care, and status of chronic vascular complications among adult patients with diabetes who presented to 71 university-affiliated diabetes outpatient clinics in Iran.

The frequency of comorbid obesity among adult people with diabetes was calculated at 42.4% for men and 52.0% for women in the NPPCD-2016 survey. Obesity and overweight in Iran are currently on a rising tide^17^. The prevalence of obesity in the general population of Iran is approximately 20%, with obesity being more frequent among women (30%) than men (17%)^18^. We observed a more gender-homogenous distribution of hypertension, with 40.3% and 38.2% estimated for men and women, respectively. The prevalence of hypertension was recently demonstrated and is gradually declining among Iranian adult community-dwellers, dropping from 25.7% in 2005 to 24.1% in 2011^19^; nevertheless little data has been made available on the frequency of obesity or hypertension among people with diabetes. Current preventive action policies should be revisited to incorporate strategies aimed at reducing the prevalence of associated comorbid cardiometabolic risk factors among Iranian adult patients with diabetes.

Around 60.5% of the NPPCD-2016 study population was on oral glucose-lowering agents. Consistent with our observation, the community-based Tehran Lipid and Glucose Study found that between 1999–2011, the use of oral glucose-lowering medications nearly doubled (from 33.4% to 60.5%) in their diabetes subcohort^20^. In NPPCD-2016, the proportions of patients on insulin monotherapy and insulin combination therapy with oral glucose-lowering medications were 11.5% and 25.1%, respectively. National diabetes reports from other countries indicate largely consistent contemporary prevalence of 15% and 19% for insulin monotherapy and combination therapy with concomitant oral glucose-lowering medicines, respectively, among people with T2D in the United Kingdom Clinical Practice Research Datalink^21^ and the frequency of 26% for insulin use among people with diabetes as reported in the Center for Disease Control and Prevention 2011 US National Diabetes Fact Sheet^22^. These findings attest that access to insulin appears to be high in Iran and is consistent with trends in the proportions of insulin use (30–40%) in most developed countries^5^. The recent widespread use of insulin in Iran is facilitated by several discernable factors; among those are the increased insurance coverage from 84% in 2010 to 95% under the May 2014 Health-Evolution Act to ensure universal model of insurance reimbursements^13^, and the free availability of imported insulin pens with the Iran Health Insurance Organization reimbursing around 90% of the costs as of 2013^13^.

The NPPCD-216 study findings confirm a subpar control of glycemic and lipid indices among the Iranian clinically-registered adult patients with different types of diabetes, with only 13.2% and 11.9% of patients achieving all preset glycemic and lipid targets. The index-specific proportions of uncontrolled hyperglycemia and hyperlipidemia among our patients were 69.9% for FPG, 72.4% for 2hPPG, 55.9% for A1C, 42.2% for triglycerides, 24.4% for total cholesterol, 40.8% for LDL-C and 63.9% for HDL-C. Between 1999–2002, 55.7% and 64.0% of the US adults with diagnosed diabetes had uncontrolled A1C and LDL-C. These numbers reduced to 47.8% and 43.2%, respectively during 2007–2010^23^. In 2007–2010, 52.5% of US patients with diabetes achieved A1C <7.0% (53 mmol/mol)^24^, rising to 54.2% by 2013^25^, and 56.2% achieved LDL-C <100 mg/dL (2.6 mmol/L)^24^. Around 78% of the present population cohort met preset blood pressure control targets; although the proportion of controlled hypertension among hypertensive patients was calculated at 43.3%. By comparison, 51.1% and 72.0% of US patients with diabetes achieved the preset targets of proper blood pressure control (defined as blood pressure cutoffs <130/80 and 140/90 mm Hg, respectively) during 2007–2010^24^. Finally, 20.2% of Iranian patients with different types of achieved all preset ABC (A1C, blood pressure and cholesterol [LDL-C]) control targets diabetes in the NPPCD-2016 survey. During 2007–2010, 18.8% of US people with diabetes achieved all ABC goals^24^ which is consistent with the Iranian estimates. Based on nationwide unpublished data from the fourth round of SuRFNCD (SuRFNCD-2011), 56.7% of the patients with diabetes did not achieve the target A1C (<7.0%), while calculated frequencies for proper hyperlipidemia control ranged from 36.9% to 39.9%. Patients with long-standing diabetes in the clinic-based cohort of Mashhad (the second most populous Metropolitan area after Tehran) had worse glycemic and lipid control, with 25.0% for A1C, 13.1% for HDL-C and 36.9% for triglycerides^26^.

We observed that hyperlipidemia and hyperglycemia control were more successful among men and women, respectively. It should be noted that owing to poorer control of diabetes-related biochemical risk factors, younger men ≤44 years old (uncontrolled FPG, 2hPPG, A1C, triglycerides, total cholesterol, and LDL-C) and older women ≥65 years old (2hPPG and A1C) represent the two high-risk groups of patients with diabetes in Iran. The former group is particularly important because of the considerable burden of disease (i.e., higher disability-adjusted life years). A recent systematic review of socioeconomic inequalities of diabetes in Iran concluded that age >55 years old (odds ratio: 5.29) and female gender (odds ratio: 1.13, 2.46) are associated with deteriorated control of diabetes^27^.

In Iran, representative statistics on the chronic vascular complications among people with diabetes have been noticeably lacking, with previous findings confined to community-, hospital- and clinic-based cohorts of local, regional or subnational levels^28–32^. Several regional studies found inconsistent frequencies for different complications, including diabetic retinopathy (30% to 40%)^30,33,34^, diabetic nephropathy (16% to 87%)^34–37^, and diabetic peripheral neuropathy (10.9% to 53%)^38,39^. These proportions were calculated in this study at 21.9% for diabetic retinopathy, 17.6% for diabetic nephropathy, and 28.0% for diabetic peripheral neuropathy. These numbers are within the range of global trends for diabetes-related chronic microvascular complications. Between 1990 and 2010, US oversaw consistent decreases in all five sentinel indicators of diabetes-related morbidity and mortality, namely myocardial infarction, hyperglycemia crisis, amputation, end-stage renal disease, and stroke^40^. Data on the prevalence of diabetic foot among Iranian patients with diabetes is noticeably scarce^13,41^, with two population-based samples of patients with T2D reporting frequencies of 0.7% and 4.0%^28,42^. A high frequency of diabetic foot in the NPPCD-2016 survey (6.2%) reflects the highest global prevalence of diabetic foot in MENA (8.6%) as a global diabetes hotspot^43^.

Cardiovascular disease is a chronic macrovascular complication and main cause of morbidity and mortality in people with diabetes^44,45^. In 2011, about one in three US adults with diabetes (age-adjusted prevalence: 33.4%) had a history of heart disease or stroke. Results from the multinational A1chieve report showed global prevalence of 27.2% for macrovascular diabetes-related complications, inclusive of myocardial infarction, angina, peripheral vascular disease, stroke, heart failure, atrial fibrillation, and left ventricular hypertrophy^43^. The lower proportion of ischemic heart disease within our study sample (24.10%) than the above-mentioned US and A1chieve studies is likely related to several factors. The US national report included data of people with diabetes who aged ≥35 years old, while the NPPCD-2016 survey reported data of patients who aged ≥18 years old. Additionally, no recorded data were available on stroke and heart diseases other than ischemia in the NPPCD database.

### Strength and limitations

This NPPCD study captured a national descriptive snapshot from the present status of diabetes and access to care in a large population of Iranian adult people with different types of diabetes who presented to the university-affiliated diabetes outpatient clinics across the country during 2015–2016 (NPPCD-2016). However, this study has several limitations. Firstly, patients presenting to the academic referral centers differ in characteristics from the entire population of people with diabetes in the community or general population and this is a possible source of selection and referral biases. Thus, the studied outcomes concerning the proportions of different types of diabetes, the associated cardiometabolic risk factors, and access to care may be different from the general population and this limitation should be carefully considered when interpreting the core findings of this study. Secondly, determinants and predictors of insufficient access to diabetes care and chronic vascular diabetes-related complications are needed in future updates from the nationwide NPPCD registry. Thirdly, there is a potential for the measurement/verification bias as a result of possible inequalities in outcome ascertainment given the large number of studied outcomes and the rigorous ascertainment of specific outcomes (i.e., IHD). Fourthly, because of the relatively low percentage of individuals with missing data on at least one of the study variables or outcomes (*n* = 542, 1.7% of the total sample size, mostly related to the random loss of such data between history taking in the clinic and entering data in the Excel datasheet), we conducted complete case analysis on data of the included patients with no missing data on any of the variables or outcomes. Although exclusion of our cases with incomplete data was unlikely to exert clinically significant alterations in the observed associations and findings, the use of strategies to handle missing data such as the multiple imputation or hot deck imputation methods could have represented more conservative alternative strategies in order to completely rule out the potential residual effects from data of excluded patients. This study is also limited by the unavailability of data on the frequencies of end-stage renal disease, blindness, amputation, stroke, peripheral vascular disease, hospitalization records or mortality, and the cross-sectional nature of associations. Accurate estimates of these conditions are required to determine the realistic burden of diabetes in Iran. A remaining issue to consider is the potential of registration bias. Many patients with T1D who are in the pediatric age range of 5–18 years old are not regularly accepted and seen in the adult diabetes outpatient clinics. These young patients with T1D are diagnosed and observed in the pediatric diabetes or pediatric endocrinology clinics because the dose titration and follow up of these patients is rather difficult and time consuming.

## Conclusions

In conclusion, close to 85% of people with diabetes who were registered in the Iranian university-affiliated adult diabetes outpatient clinics were diagnosed with T2D. In addition, women constituted 66.5% of all patients included in the NPPCD-2016 study. Men with diabetes had higher frequencies of hypertension and smoking, while women with diabetes were more frequently obese. Despite the high access to diabetes medications and wide insulin coverage, the current quality of national diabetes control remains subpar, compared with other areas of the world and consensus clinical targets. Men had better control of hyperlipidemia and women were more successful in proper control of hyperglycemia. Younger men and older women comprise two high-risk groups of people with diabetes in Iran, owing to the suboptimal control of cardiometabolic related plasma risk factors. Proportions of chronic vascular complications remain high in both genders. Based on these findings, preventative strategies of NPPCD on targeting different types of diabetes, related comorbidities and complications should be revisited to incorporate primordial prevention and lifestyle interventions as means of bridging the translation gap between largely adequate therapeutic resources^46,47^ and universally subpar metrics of diabetes control in Iran.

### What is already known on this subject

- Diabetes is a major risk factor for cardiovascular disease. The prevalence and burden of diabetes is high and is concurrently increasing in Iran, growing by 35% among the general adult population of Iran during 2005–2011.

### What this study adds?

- This study which was sanctioned by the National Program for Prevention and Control of Diabetes (NPPCD) investigated the estimated proportions of different types of diabetes in addition to the associated modifiable and non-modifiable cardiometabolic risk factors, control, medication use, and chronic vascular complications among Iranian adult people with diabetes referred to country’s academic tertiary-care diabetes outpatient clinics.
- This prospective analysis on 30,202 adult people with clinically-diagnosed diabetes is the inaugural report from the nationwide NPPCD database on the status of diabetes care in Iran (NPPCD-2016). The analyzed data were registered in the diabetes outpatient clinics of university-affiliated hospitals across the country. The STROBE guidelines were used for reporting the results of this study.
- The proportions of type 1 diabetes, types 2 diabetes, and other types of diabetes were calculated at 11.4%, 85.5%, 1.8%, and 1.3%, respectively among the clinically-registered adult patients with diabetes in Iran.
- The proportions of drug-naivety, use of oral agents, insulin monotherapy and insulin combination therapy were 2.9%, 60.5%, 11.5%, and 25.1%, respectively.
- More than 97% of Iranian adults with a diagnosis of diabetes consume oral glucose-lowering agents, inject insulin or use a combination of medications and insulin. However, as low as 13.2%, 11.9% and 43.3% of these patients have controlled hyperglycemia, hyperlipidemia and hypertension, respectively. In total, the proportion of achieving the preset ABC (A1C, blood pressure and cholesterol [LDL-C]) control targets was calculated at 20.2% in the study population. These data indicate that despite the wide availability of diabetes medication and insulin coverage in Iran, the proportions of proper hyperglycemia, hyperlipidemia and hypertension control (especially for younger men and older women) remains subpar among Iranian adult patients with diabetes presenting to country’s university-affiliated diabetes outpatient clinics.
- The proportions of diabetic retinopathy, diabetic nephropathy, diabetic peripheral neuropathy, diabetic foot ulcers, and ischemic heart disease within the population of patients in tertiary care centers were 21.9%, 17.6%, 28.0%, 6.2%, and 23.9%, respectively. Echoing the concerning status of the Middle East and the North Africa (MENA) region, the frequencies of diabetes-related chronic vascular complications, in particular the diabetic foot, is relatively high among adult patients with diabetes admitting to tertiary care centers in Iran.
- Preventative strategies of NPPCD on targeting different types of diabetes, related comorbidities and complications should be revisited to incorporate primordial prevention and changes in lifestyle as means of bridging the translation gap between largely adequate resources and universally subpar metrics of diabetes control in Iran.

## Electronic supplementary material

Supplementary Material
