# Supplementary material for: Diabetes in Iran: Prospective Analysis from First Nationwide Diabetes Report of National Program for Prevention and Control of Diabetes (NPPCD-2016)
Source: Sci Rep. 2017 Oct 18;7:13461. doi: 10.1038/s41598-017-13379-z (PMC5647418; doi:10.1038/s41598-017-13379-z)
Supplement: Supplementary file 1 — Supplementary Material [file 41598_2017_13379_MOESM1_ESM.pdf]

Diabetes in Iran: Prospective Analysis from First Nationwide Diabetes Report of National Program for Prevention and Control of Diabetes (NPPCD-2016)

Running Head: Diabetes Care in Iran

Alireza Esteghamati[Ϯ]*, M.D.; Bagher Larijani[ǂ], M.D., Mohammad Haji Aghajani[Ω], M.D., Fatemeh Ghaemi[α], M.D.; Jamshid Kermanchi[β], M.D.; Ali Shahrami[Ω], M.D.; Mohammad Saadat[Ϯ], M.D., M.P.H.; Ensieh Nasli Esfahani[ǂ], M.D.; Morsaleh Ganji[Ϯ], M.D.; Sina Noshad[Ϯ], M.D., M.P.H.; Elias Khajeh[Ϯ], M.D., M.P.H.; Alireza Ghajar[Ϯ], M.D.; Behnam Heidari, [Ϯ] M.D., M.P.H.; Mohsen Afarideh[Ϯ] M.D., M.P.H.; Jeffrey I. Mechanick[¶], M.D., F.A.C.P., F.A.C.E., F.A.C.N., E.C.N.U.; Faramarz Ismail- Beigi[‡],, M.D., Ph.D.

Ϯ Endocrinology and Metabolism Research Center (EMRC), Vali-Asr Hospital, School of Medicine, Tehran University of Medical Sciences, Tehran, Iran

ǂ Diabetes Researcher Center, Endocrinology and Metabolism Research Institute, Tehran University of Medical Sciences, Tehran, Iran

Ω Shahid Beheshti University of Medical Sciences, Tehran, Iran

α Diabetes Program, Deputy of Health, Ministry of Health and Medical Education (MOHME), Tehran, Iran

β Deputy of Curative Affairs, Ministry of Health and Medical Education (MOHME), Tehran, Iran

¶ Division of Endocrinology, Diabetes, and Bone Disease, Icahn School of Medicine at Mount Sinai, New York, New York

‡ Department of Medicine, Biochemistry, Physiology and Biophysics, Division of Clinical and Molecular Endocrinology, Case Western Reserve University, Cleveland, Ohio

*Correspondence should be addressed to Professor Alireza Esteghamati, MD (esteghamati@tums.ac.ir), Endocrinology and Metabolism Research Center, Vali-Asr Hospital, School of Medicine, Tehran University of Medical Sciences, P.O. Box 13145-784, Tehran, Iran

Fax: (+9821)-64432466

Tel: (+9821)-88417918

SUPPLEMENTARY MATERIAL

Results

In people with T2D, people from older age categories displayed higher proportions of hypertension (gender-specific *p* for trend values < 0.001), older men had a lower frequency of obesity and smoking habit (*p* for trend values = 0.001 and 0.005), and older women were more frequently smokers (*p* for trend value = 0.009). Higher durations of disease were noted among all patients with T2D and hypertension (gender-specific *p* for trend value < 0.001). Among patients with T2D, Men without obesity (*p* for trend value = 0.002) and women who smoked tobacco (*p* for trend value = 0.011) had increased duration of disease compared to their obese and nonsmoker counterparts, respectively.

For patients with T2D, the rates (95% CIs) of drug-naivety, use of oral glucose-lowering agents, insulin monotherapy and insulin combination therapy with oral agents were 2.2 % (2.0% to 2.4%), 68.1% (67.5% to 68.7%), 6.6% (6.3% to 6.9%) and 22.8% (22.3% to 23.3%), respectively (**SUPPLEMENTARY TABLE S1**).

In people with T2D, women from the older age groups were less frequently drug-naive (*p* for trend value < 0.001) and more regularly received combined regimen of insulin and oral agents (*p* for trend value = 0.001). Significantly higher rates for the use of oral glucose-lowering agents and insulin monotherapy (*p* for trend values = 0.047 and 0.025, respectively) and lower rates of drug-naivety (*p* for trend value < 0.001) were observed among younger men (**SUPPLEMENTARY TABLE S1**). In the group of patients with T2D, men and women with long-standing diabetes (> 20 years) had significantly higher rates of insulin monotherapy (*p* for

trend value < 0.001) or insulin therapy in combination with glucose-lowering medications (*p* for trend value < 0.001) and lower rates of drug-naivety (*p* for trend value < 0.001) or glucose-lowering medication use (*p* for trend value < 0.001), compared to those with a shorter duration of diabetes (< 10 years) (**SUPPLEMENTARY TABLE S2**).

In the subset of patients with T2D, men had better overall control of lipid targets than women, although the overall control of glycemic targets was comparable between men and women. Older men with T2D had significantly better overall control of glycemic and lipid indicators than their younger counterparts. Older women with T2D also had better overall control of hyperlipidemia than younger women with T2D; however the overall glycemic control was better achieved in younger women with T2D (**SUPPLEMENTARY TABLE S1**). Poorer control of hyperglycemia was observed in men and women with T2D who had higher durations of disease (gender-specific *p* for trend values < 0.001) compared to their counterparts with more recent diagnoses (**SUPPLEMENTARY TABLE S2**). Better control of hyperlipidemia was present in men with longer disease duration (*p* for trend value < 0.001). In contrast, women with higher duration of T2D had a worse global control of hyperlipidemia compared to their peers with more recent diagnoses (*p* for trend value < 0.001, **SUPPLEMENTARY TABLE S2**).

In the subgroup of patients with T2D, older patients had significantly higher proportions of diabetic retinopathy, diabetic nephropathy, diabetic peripheral neuropathy, ischemic heart disease, and diabetic foot (*p* for trend values for all complications in both genders < 0.001) (**SUPPLEMENTARY TABLE S1**). Men and women with longer duration of T2D had

significantly higher frequencies of diabetic retinopathy, diabetic nephropathy, diabetic peripheral neuropathy, ischemic heart disease, and diabetic foot ulcers (gender-specific *p* for trend values for each complication < 0.001) (**SUPPLEMENTARY TABLE S2**).

Conclusions

Low rates of drug-naive Iranian men (3.5%) and women (2.5%) with diabetes contrasts with observations from other developed regions. Around 20% of U.K. adult population with T2D was on diet-control management from 2007 to 2009 (1). The rate of US diabetes diet-control was 16% according to the 2011 U.S. National Diabetes Fact Sheet (2) and decreased to 14.4% in 2014 (3); however, these rates are substantially higher than those reported here. In addition, the current database does not distinguish between people who engaged in lifestyle modification (e.g., physical activity and restricted caloric intake) or those who simply refused to seek treatment/were denied of medications for their diabetes (i.e., nontreatment). As such, this study may overestimate the number of people with the tag of "lifestyle intervention" for diabetes management. Many cultural, socioeconomic and psychosocial related risk factors pertaining to the background of Iranian Society contribute to these alarmingly low rates of medication-naive people with diabetes in Iran.

Patients with < 10 years of diabetes duration had comparable control of preset glycemic targets to patients with > 20 years since their time of diagnosis (ANOVA Post-hoc Bonferroni *p* values for FPG, 2hPPG and A1C = 0.405, 0.682 and 0.980), with both groups showing inferior glycemic control to people with a diagnosis of diabetes during the past 10-20 years. Several factors contribute to this suddenly compromised diabetes control in people with > 20 years of

diabetes duration who are routinely required to move on to insulin injection for diabetes management. Aside from cultural, socioeconomic, and psychological factors, a dramatic drop in the adherence to medications, despite their high availability, with progressive years of chronic diabetes management is noteworthy. In particular, poor adherence with prescribed insulin regimens in the forms of “injection fear”, “hardship from insulin injection” or “high cost of insulin therapy” is a recurring theme among Iranian patients with diabetes (4) and has seemingly stagnated the improving status of diabetes control, despite substantial progressions in terms of insulin coverage in the country.

Importantly, the control of hyperlipidemia showed little variations by age or duration of diabetes. We believe slightly better control of lipid targets in higher age groups or in those with longer courses of disease are partially mediated by the systematic initiation of statins in older patients with diabetes or in those with significantly larger amount of time since their diagnosis of diabetes. Unlike hyperglycemia, the control of lipid indices is achieved more readily with the use of only medications and thus, has a generally higher rate of success.

1. Holden S, Gale E, Jenkins-Jones S, Currie C. How many people inject insulin? UK estimates from 1991 to 2010. Diabetes, Obesity and Metabolism. 2014;16(6):553-9.

2. Control CfD. Prevention (2011) National Diabetes Fact Sheet: National Estimates and General Information on Diabetes and Prediabetes in the United States. Department of Health and Human Services, Centers for Disease Control and Prevention.

3. Control CfD, Prevention. National diabetes statistics report: estimates of diabetes and its burden in the United States, 2014. Atlanta, GA: US Department of Health and Human Services. 2014; 2014.

4. Ghadiri-Anari A, Fazaelipoor Z, Mohammadi SM. Insulin refusal in Iranian patients with poorly controlled type 2 diabetes mellitus. Acta Medica Iranica. 2013;51(8):567.

SUPPLEMENTARY TABLE S1 Proportions of chronic microvascular and macrovascular complications and meeting preset glycemic and lipid targets according by age in clinically-registered Iranian adult patients with type 2 diabetes.

		Frequency	Retinopathyϕǂ	Nephropathy*ϕǂ	Neuropathy*ϕǂ	Ischemic Heart Disease *ϕǂ	Diabetic Foot *ǂ	Treatment of Diabetes*ϕǂ				All Glycemic Indices Control ϕǂ	All Lipid Indices Control *ϕǂ
								Drug-Naïve	Oral Agent	Insulin	Combination		
Gender	Age (years)	Number (%)	Proportion	Proportion	Proportion	Proportion	Proportion	Proportion	Proportion	Proportion	Proportion	Proportion	Proportion
			95% CI	95% CI	95% CI	95% CI	95% CI	95% CI	95% CI	95% CI	95% CI	95% CI	95% CI
Women	≤ 44	1,642 (6.4%)	10.20%	10.50%	19.20%	10.40%	3.20%	3.50%	66.10%	10.20%	20.10%	14.40%	8.60%
			8.74 - 11.66	9.02 - 11.98	17.29 - 21.11	8.92 - 11.88	2.35 - 4.05	2.61 - 4.39	63.81 - 68.39	8.74 - 11.66	18.16 - 22.04	12.70 - 16.10	7.24 - 9.96
	45 to 64	11,657 (45.1%)	20.70%	15.50%	29.40%	22.40%	5.00%	1.40%	68.90%	5.30%	24.40%	12.90%	9.10%
			19.96 - 21.44	14.84 - 16.16	28.57 - 30.23	21.64 - 23.16	4.60 - 5.40	1.19 - 1.61	68.06 - 69.74	4.89 - 5.71	23.62 - 25.18	12.29 - 13.51	8.58 - 9.62
	≥ 65	3,901 (15.1%)	26.50%	20.00%	33.30%	33.70%	6.10%	1.00%	68.60%	7.20%	23.10%	13.20%	10.30%
			25.12 - 27.88	18.74 - 21.26	31.82 - 34.78	32.22 - 35.18	5.35 - 6.85	0.69 - 1.31	67.14 - 70.06	6.39 - 8.01	21.78 - 24.42	12.14 - 14.26	9.35 - 11.25
	Total	17,200 (66.6%)	21.10%	16.00%	29.30%	23.80%	5.10%	1.50%	68.60%	6.20%	23.70%	13.10%	9.40%
			20.49 - 21.71	15.45 - 16.55	28.62 - 29.98	23.16 - 24.44	4.77 - 5.43	1.32 - 1.68	67.91 - 69.29	5.84 - 6.56	23.06 - 24.34	12.60 - 13.60	8.96 - 9.84
Men	≤ 44	891 (3.5%)	10.20%	12.70%	17.10%	8.30%	5.40%	7.90%	65.10%	7.90%	19.20%	11.30%	11.10%
			8.21 - 12.19	10.51 - 14.89	14.63 - 19.57	6.49 - 10.11	3.92 - 6.88	6.13 - 9.67	61.97 - 68.23	6.13 - 9.67	16.61 - 21.79	9.22 - 13.38	9.04 - 13.16
	45 to 64	5,031 (19.5%)	20.60%	16.50%	23.10%	22.10%	7.10%	3.20%	68.10%	6.90%	21.80%	12.30%	16.40%
			19.48 - 21.72	15.47 - 17.53	21.94 - 24.26	20.95 - 23.25	6.39 - 7.81	2.71 - 3.69	66.81 - 69.39	6.20 - 7.60	20.66 - 22.94	11.39 - 13.21	15.38 - 17.42
	≥ 65	2,697 (10.4%)	24.40%	22.80%	28.40%	34.30%	7.50%	2.30%	69.20%	8.40%	20.10%	12.80%	18.10%
			22.78 - 26.02	21.22 - 24.38	26.70 - 30.10	32.51 - 36.09	6.51 - 8.49	1.73 - 2.87	67.46 - 70.94	7.35 - 9.45	18.59 - 21.62	11.54 - 14.06	16.65 - 19.55
	Total	8,619 (33.4%)	20.70%	18.10%	24.10%	24.50%	7.00%	3.40%	68.10%	7.50%	21.00%	12.30%	16.50%
			19.84 - 21.56	17.29 - 18.91	23.20 - 25.00	23.59 - 25.41	6.46 - 7.54	3.02 - 3.78	67.12 - 69.08	6.94 - 8.06	20.14 - 21.86	11.61 - 12.99	15.72 - 17.28
Total		25819 (100%)	20.90%	16.70%	27.60%	24.10%	5.70%	2.20%	68.40%	6.60%	22.80%	12.90%	11.60%
			20.40 - 21.40	16.25 - 17.15	27.05 - 28.15	23.58 - 24.62	5.42 - 5.98	2.02 - 2.38	67.53 - 68.67	6.30 - 6.90	22.29 - 23.31	12.49 - 13.31	11.21 - 11.99

95% CI: 95% confidence interval

* Denotes statistically significant difference between men and women (p value < 0.05).

ϕ Denotes statistically significant difference across the age groups of women (p value < 0.05).

Ŧ Denotes statistically significant difference across the age groups of men (p value < 0.05).

SUPPLEMENTARY TABLE S2 Proportions of chronic microvascular and macrovascular complications and meeting the preset glycemic and lipid targets according to the duration of disease in clinically-registered Iranian adult patients with type 2 diabetes.

		Frequency	Retinopathyϕǂ	Nephropathy*ϕǂ	Neuropathy*ϕǂ	Ischemic Heart Disease *ϕǂ	Diabetic Foot *ϕǂ	Treatment of Diabetes*ϕǂ				All Glycemic Indices Control ϕǂ	All Lipid Indices Control *ϕǂ
								Drug-Naïve	Oral Agent	Insulin	Combination		
Gender	Duration (years)	Number (%)	Proportion	Proportion	Proportion	Proportion	Proportion	Proportion	Proportion	Proportion	Proportion	Proportion	Proportion
			95% CI	95% CI	95% CI	95% CI	95% CI	95% CI	95% CI	95% CI	95% CI	95% CI	95% CI
Women	< 10	10,151 (39.7%)	15.40%	14.80%	26.80%	20.20%	4.20%	2.20%	76.70%	4.40%	16.70%	16.40%	8.50%
			14.70 – 16.10	14.11 - 15.49	25.94 - 27.66	19.42 - 20.98	3.82 - 4.58	1.91 – 2.49	75.88 - 77.52	4.00 - 4.80	15.97 - 18.43	15.68 - 17.12	7.96 - 9.05
	10 to 20	5,225 (20.4%)	27.60%	17.80%	32.90%	27.40%	5.60%	0.60%	59.80%	8.10%	31.50%	8.20%	10.40%
			26.38 - 28.81	16.76 – 18.84	31.63 - 34.17	26.19 - 28.61	4.98 - 6.22	0.39 - 0.81	58.47 - 61.13	7.36 - 8.84	30.24 - 32.76	7.46 - 8.94	9.67 - 11.33
	> 20	1,688 (6.6%)	35.40%	18.30%	33.40%	35.30%	9.20%	0.50%	45.70%	10.90%	42.90%	8.10%	13.20%
			33.12 - 37.68	16.64 - 20.14	31.15 – 35.65	33.05 - 37.58	7.82 - 10.58	0.16 - 0.84	43.32 - 48.08	9.41 - 12.37	40.54 - 45.27	6.80 - 9.40	11.59 - 14.81
	Total	17,064 (66.7%)	21.10%	16.10%	29.30%	23.90%	5.10%	1.50%	68.50%	6.20%	23.80%	13.10%	9.40%
			20.49 - 21.72	15.55 - 16.65	28.62 - 29.98	22.26 - 24.54	4.77 - 5.43	1.32 - 1.68	67.80 - 69.20	5.84 - 6.56	23.16 - 24.44	12.59 - 13.62	8.96 - 9.84
Men	< 10	5,511 (21.5%)	15.40%	16.70%	21.90%	21.30%	5.70%	4.70%	74.20%	5.50%	15.60%	15.00%	15.60%
			14.44 - 16.36	15.72 - 17.68	20.81 - 22.99	20.22 - 22.38	5.09 - 6.31	4.14 - 5.26	73.04 - 75.36	4.90 - 6.10	14.64 - 16.56	14.06 - 15.92	14.64 - 16.56
	10 to 20	2,208 (8.6%)	29.20%	20.60%	27.80%	29.10%	8.30%	1.00%	59.10%	10.40%	29.60%	7.00%	18.20%
			27.30 - 31.10	18.91 - 22.29	25.93 - 29.67	27.21 - 30.99	7.15 - 9.45	0.58 - 1.42	57.05 - 61.15	9.13 - 11.67	27.70 - 31.50	5.94 - 8.06	16.59 – 19.81
	> 20	817 (3.2%)	35.00%	21.40%	30.50%	34.40%	12.70%	1.00%	50.20%	13.80%	35.00%	8.30%	20.60%
			31.73 – 38.27	18.59 - 24.21	27.34 – 38.58	31.14 - 37.66	10.42 - 14.98	0.32 - 1.68	46.57 - 53.63	11.43 - 16.17	31.73 - 38.27	6.42 - 10.18	17.83 - 23.37
	Total	8,536 (33.3%)	20.90%	18.20%	24.20%	24.50%	7.10%	3.40%	68.00%	7.60%	21.10%	12.30%	16.60%
			20.04 - 21.76	17.38 - 19.02	23.29 - 25.11	23.59 - 25.41	6.56 - 7.64	3.02 - 3.78	67.01 - 68.99	7.04 - 8.16	20.33 - 21.97	11.60 - 13.00	15.81 - 17.39
Total		25,600 (100%)	21.00%	16.80%	27.60%	24.10%	5.80%	2.10%	68.30%	6.60%	22.90%	12.80%	11.70%
			20.50 - 21.50	16.34 - 17.26	27.05 - 28.15	23.58 - 24.62	5.51 - 6.09	1.92 - 2.28	67.73 - 68.87	6.30 - 6.90	22.39 - 23.41	12.39 - 13.21	11.31 - 12.09

95% CI: 95% confidence interval

* Denotes statistically significant difference between men and women (*p* value < 0.05).

ϕ Denotes statistically significant difference across different groups of diabetes duration in men (*p* value < 0.05).

Ŧ Denotes statistically significant difference across different groups of diabetes duration in men (*p* value < 0.05).
